# 3,4-Dihydroxybenzenesulfonyl-Functionalized Polyethyleneimine for Uranium Chelation

**DOI:** 10.3390/polym17162256

**Published:** 2025-08-21

**Authors:** Kai Liang, Sifan Liu, Fan Zhang, Wenjin Cui, Ying Tian, Shuchen Liu, Lin Wang

**Affiliations:** 1 School of Pharmacy, Guangdong Pharmaceutical University, Guangzhou 510006, China; a2724963261@163.com; 2 Beijing Institute of Radiation Medicine, Beijing 100850, China; 18146539471@163.com (S.L.); zhangfan201103@163.com (F.Z.); cuiwj112233@163.com (W.C.); hq6106@aliyun.com (Y.T.); 3 School of Pharmacy, Henan University, Kaifeng 475001, China; 4 College of Chemistry and Life Science, Beijing University of Technology, Beijing 100124, China

**Keywords:** uranium chelator, polyethyleneimine, siderophores

## Abstract

3,4-dihydroxybenzenesulfonyl-functionalized polyethyleneimine (PS), a novel polymeric chelator, was synthesized by conjugating 3,4-dihydroxybenzenesulfonyl (CAM) groups with branched polyethyleneimine (BPEI, MW = 600 Da) via N-acylation. PS demonstrated a high uranium adsorption capacity of 78.08% at a concentration of 4 mg/mL, accompanied by significant selectivity over competing ions such as Ca^2+^, Zn^2+^, and Cu^2+^. Notably, in competitive adsorption experiments, PS exhibited a uranium adsorption rate of 59.49%, which was 3.95 times higher than that of calcium (15.06%) in the Ca^2+^ system. Cytotoxicity assays revealed enhanced biocompatibility (IC_50_ = 86.98 μg/mL), surpassing CaNa_3_-DTPA 3.7-fold. In a uranium exposure model (200 μg/mL), PS significantly improved cell survival rates and reduced intracellular uranium levels by 77.37% (immediate administration) and 64.18% (delayed administration). These findings establish PS as a potent and safe polymeric chelator for uranium decorporation, offering a promising strategy for mitigating the hazards of radioactive materials.

## 1. Introduction

With the wide application of nuclear technology in industry, agriculture, medicine, military, and many other fields, radiation has also become a technological double-edged sword. The trans-media migration mechanism of radionuclide uranium in the environmental–biological interface exacerbates pollution hazards. Uranium, in the form of uranyl ions (UO_2_^2+^), has a long-term presence and enters the human body through the bioconcentration effect of the food chain [1]. After entering the body, it mainly deposits in the kidneys, bones, and liver, is difficult to discharge, and triggers irreversible internal irradiation damage and metal toxicity effects [2,3].

Currently, the treatment for uranium pollution is facing the double technical bottleneck of environmental remediation and in vivo excretion promotion. Traditional chelating agents (such as DTPA) in seawater media experience reduced uranium adsorption capacity due to the interference of competing ions [4]. In vivo, they are limited by the coordination mode and find it difficult to achieve the ideal effect of promoting uranium excretion. Their use is prone to cause kidney damage and other toxic side effects [5,6]. It is challenging to balance the coordination stability and biosafety of this linear small molecule chelator in complex media, which seriously limits its application in cross-scenario uranium contamination management. Given the many limitations of traditional chelating agents in treating uranium contamination, researchers have begun to look to polymeric materials that offer unique advantages.

Polymers offer distinct advantages over traditional small molecule chelators, primarily due to their high chelation capacity and unique biodistribution characteristics [7]. The increased number of chelation sites per unit area in polymers enhances complexation efficiency, while their larger size leads to mechanical retention by major organs, facilitating indirect targeting and improved drug delivery [8].

Branched polyethyleneimine (BPEI), a water-soluble polymer, stands out due to its high solubility [9], high capacity [10], biocompatibility [11], and indirect targeting ability [12]. These properties make it promising for diverse applications. The polyamino structure of BPEI allows surface modification via reaction with epoxide groups, acids, acyl chloride, and isocyanate. This modified BPEI can remove in vitro contaminants and promote in vivo nuclide excretion. Moreover, as a DTPA polymer derivative, BPEI has abundant chelating sites. These sites can be targeted to specific areas such as the lungs, bones, liver, or kidneys, potentially revolutionizing the field of decorporation chelating agents. Specifically, a 600 Da BPEI molecule provides approximately 14 amine coordination sites, as confirmed by potentiometric titration [13]. Molecular dynamics simulations reveal that the branched architecture of BPEI offers superior conformational flexibility compared to linear PEI (LPEI), enabling synergistic interactions between secondary and tertiary amine groups that enhance uranyl complex stability [7]. Crucially, low molecular weight BPEI (e.g., 600 Da) shows enhanced cellular internalization and reduced cytotoxicity. This is because its lower surface charge density weakens electrostatic interactions with plasma membranes, preserving membrane integrity [14]. Moreover, cytotoxicity assays indicate that 600 Da BPEI only affects cell viability at concentrations much higher than those needed for therapeutic effects [15]. This significant safety window between effective dose and cytotoxic threshold strongly supports the potential of BPEI as a versatile carrier platform for pharmaceutical development. Importantly, the preserved cellular viability at pharmacologically relevant concentrations aligns with previous optimized BPEI-based delivery systems showing enhanced biocompatibility through structural modifications [16]. In summary, BPEI demonstrates significant potential in drug development due to its multifaceted advantages. Its favorable biocompatibility and low toxicity profile further solidify its position as an excellent candidate for decorporation agent development. For example, pioneering work by Lahrouch et al. established methylcarboxylated polyethyleneimine (PEI-MC) as an effective uranium decorporation agent through multi-technique characterization (ICP-MS, FT-IR), demonstrating 0.47 mg U(VI)/mg loading capacity via mono- and bi-dentate coordination [8]. Subsequent research on polyethyleneimine-methylphosphonate (PEI-MP) further demonstrated actinide-binding capabilities, with U(VI)- and Th(IV)-loading capacities reaching 0.56–0.80 mg/mg and 0.15–0.20 mg/mg, respectively [17]. Collectively, these findings highlight the transformative potential of polymeric chelators in advancing treatments for internal radionuclide contamination.

Simultaneously, siderophores, with high Fe^3+^ binding ability, can also chelate UO_2_^2+^ due to their similar coordination configurations. Given their high selectivity, low toxicity, and efficiency, siderophores are ideal chelating ligands [18]. This study innovatively designs a novel uranium chelator, PS, by grafting siderophore-specific CAM groups onto BPEI (MW = 600 Da) via N-acylation. This design aims to combine the high chelation capacity of BPEI with the high selectivity of siderophores, thereby addressing the limitations of current decorporation agents and enhancing the efficiency and safety of uranium removal. This design leverages BPEI’s polyamine sites for uranium enrichment and passive targeting, while siderophore ligands precisely recognize the uranyl ion’s octahedral configuration, forming a multi-level chelation network. The optimized 600 Da BPEI balances cellular uptake and biocompatibility, synergistically enhancing chelation capacity and biocompatibility, offering new insights for developing chelating agents.

## 2. Materials and Methods

### 2.1. Materials

3,4-Dimethoxybenzenesulfonyl chloride (98%) was purchased from Shanghai Titan Technology Co., Ltd. (Shanghai, China). BPEI (MW = 600 Da, 1800 Da, 10,000 Da), triethylamine (TEA, 99.5%), and boron tribromide solution (1.0 M in CH_2_Cl_2_) were obtained from Beijing Inokai Technology Co., Ltd. (Beijing, China). Copper(II) chloride, anhydrous zinc chloride, ferric chloride, and anhydrous calcium chloride were purchased from Anhui Senrise Technology Co., Ltd. (Hefei, China). Regenerated cellulose dialysis membranes (MWCO 500 Da) were supplied by Xi’an Youbo Biotechnology Co., Ltd. (Xi’an, China). Uranyl acetate dihydrate [UO_2_(CH_3_COO)_2_·2H_2_O] and CaNa_3_-DTPA (100 mg/mL pharmaceutical grade) were sourced from Shanghai Jizhi Biochemical Technology Co., Ltd. (Shanghai, China), and the Military Medical Research Institute (Beijing, China), respectively. All other analytical grade reagents were procured from Sinopharm Chemical Reagent Co., Ltd. (Shanghai, China) and used without further purification.

For cell experiments, U(VI) stock solution (20 mg/mL) was prepared by dissolving UO_2_(CH_3_COO)_2_·2H_2_O in deionizing water, after the U(IV) working solution of 320 μg/mL was prepared by diluting the stock solution with deionized water and filtering through the microporous membrane with 0.22 μm pore diameter before use.

### 2.2. Analytical Techniques

^1^H NMR spectra were recorded on a Bruker ECA-400 MHz (Bruker Co., Billerica, MA, USA) spectrometer with TMS as an internal standard. Chemical shifts were in ppm (δ), and coupling constants (J) were reported in Hertz (Hz). Fourier transform infrared spectra (FT-IR) were recorded on a Nicolet iS20 spectrometer (Thermo Fisher Scientific, Waltham, MA, USA) in the 4000–400 cm^−1^ range at 4 cm^−1^ resolution; samples were prepared as KBr pellets and measured in transmission mode. Scanning electron microscope (SEM) images were recorded at 0.02–30 kV on a ZEISS GeminiSEM 300 (Carl Zeiss AG, Oberkochen, Germany) equipped with a Schottky field emission electron gun. In vitro, absorbance was measured with a full-wavelength enzyme labeler (Multiskan SkyHigh, Thermo Fisher Scientific, Waltham, MA, USA). Uranium concentration was determined by inductively coupled plasma mass spectrometry (ICP-MS) on a Thermo Fisher Scientific iCAP RQ spectrometer (Thermo Fisher Scientific, Waltham, MA, USA); samples were prepared by direct dilution with 2% HNO_3_ and analyzed in kinetic-energy-discrimination (KED) mode with ^209^Bi as the internal standard.

### 2.3. Synthesis of PS

**Figure 1:**
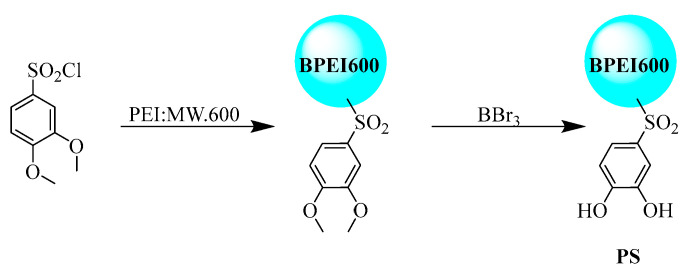
Two-step synthesis of sulfonamide-functionalized PS from PEI.

The reaction was initiated by dissolving 3,4-dimethoxybenzenesulfonyl chloride (1 g, 4.22 mmol) in tetrahydrofuran (5 mL), which was subsequently added dropwise to a chilled (0 °C) THF solution (5 mL) containing PEI (0.182 g) under continuous stirring. Triethylamine (0.05 mL) and 4-dimethylaminopyridine (2 mg) were introduced as catalytic additives. Vigorous gas evolution accompanied by precipitate formation was observed during the 4 h reaction period [19]. The resultant mixture was diluted with deionized water (40 mL) to solubilize precipitated byproducts, followed by purification through dialysis (regenerated cellulose membrane, MWCO 500 Da) until complete removal of low-molecular-weight species was confirmed. Filtration yielded an intermediate product (0.487 g) that was subsequently dissolved in dichloromethane (10 mL). Boron tribromide (12 mL) was added dropwise to the chilled (0 °C) solution over 30 min, maintaining vigorous agitation for 6 h to ensure complete demethylation [20]. The reaction was quenched with methanol (5 mL) until gas evolution ceased, followed by solvent removal via rotary evaporation under reduced pressure. The residue was reconstituted in methanol, diluted with an equal volume of water, and subjected to secondary dialysis (MWCO 500 Da). Final purification involved repeated cold water washes (3 × 20 mL) followed by vacuum desiccation, yielding the target compound PS as a hygroscopic solid (0.048 g, 9.85% overall yield based on intermediate product mass) (Figure 1).

### 2.4. Adsorption of Uranium

#### 2.4.1. Adsorption Capacity

Different masses (8, 4, 2, 1, and 0.5 mg) of PS were precisely weighed and introduced into individual 2 mL aqueous solutions containing 50 mg/L UO_2_(CH_3_COO)_2_·2H_2_O to establish adsorption systems. The systems were then transferred to a thermostatic shaker and agitated at 150 rpm for 24 h at 37 °C to achieve adsorption equilibrium. Subsequently, the mixtures were centrifuged at 12,000 rpm for 15 min to separate the adsorbent from the aqueous phase. The supernatant was carefully collected, filtered through a 0.22 μm membrane to remove residual particulates, and analyzed for residual U(VI) concentration using ICP-MS. All experiments were conducted in triplicate with parallel blank controls containing no PS to ensure reproducibility and eliminate background interference.

#### 2.4.2. Competing Adsorption Experiments

Selectivity experiments were conducted under conventional single-metal ion conditions to evaluate the adsorption performance. The experimental setup involved preparing aqueous solutions containing 50 mg/L uranium along with 250 mg/L of individual competing metal ions (Ca^2+^, Zn^2+^, Fe^3+^, Cu^2+^). For each trial, 4 mg of adsorbent material was added to 2 mL of the metal-containing uranyl solution. The adsorption systems were maintained at 37 °C in a thermostatic shaker operating at 150 rpm for 24 h to ensure equilibrium attainment. Following the adsorption phase, the mixtures underwent centrifugation at 12,000 rpm for 15 min to separate the adsorbent from the liquid phase. The supernatant was subsequently collected and analyzed for residual U(VI) concentrations using ICP-MS. All experiments were conducted in triplicate with appropriate blank controls to ensure reproducibility and minimize interference.

### 2.5. Cell Line and Culture

The NRK-52E cell line (organism: kidney, rat, ATCC^®^, CRL-1571 DSMZ, CL-0174) was purchased from Wuhan Pricella Biotechnology Co., Ltd. (Wuhan, Hubei, China) and was cultured in a complete medium containing DMEM (PM150210), 5% FBS (164210-50), and 1% P/S (PB180120) in a humidified atmosphere of 5% CO_2_ at 37 °C. The cells were propagated every two~three days.

#### Cytotoxicity Assay

A cytotoxicity assay was conducted using the Cell Counting Kit-8 (CCK-8), provided by Applygen Technologies Co., Ltd., Beijing, China. The cytotoxic effects of the compounds were evaluated by determining the concentration that reduced cell growth by 50%, known as the IC_50_ value. CaNa_3_-DTPA served as a positive control in this assay. NRK-52E cells in the logarithmic growth phase were seeded into 96-well plates at a density of 5 × 10^3^ cells per well. The plates were cultured in an incubator at 37 °C with 5% CO_2_ for 24 h. The original medium was discarded, and the cells were treated with a medium containing different test compounds, whereas the blank control group received only culture media. This was followed by a further 48 h of incubation. Subsequently, 10 μL of CCK-8 solution was added to each well, and the plates were incubated for 1–2 h. The absorbance of each well at 450 nm was measured using a full-wavelength enzyme labeler. The formula used to determine the survival rate was as follows: [OD experimental]/[OD blank] 100%.

### 2.6. Effect of Chelating Agents on U(VI)-Induced NRK-52E Cells Injury

The therapeutic effects of the chelating agents were evaluated by modeling a 50% reduction in cell viability induced by U(VI). NRK-52E cells, in the logarithmic growth phase, were plated in 96-well plates at a density of 5 × 10^3^ cells per well. The plates were incubated at 37 °C in an atmosphere containing 5% CO_2_ for 24 h. After removal of the culture medium, the cells were concurrently exposed to 200 μg/mL UO_2_(CH_3_COO)_2_·2H_2_O and 10~80 μg/mL of the chelating agent for 48 h. Groups exposed to U(VI) only were treated with UO_2_(CH_3_COO)_2_·2H_2_O, while the blank control group was treated with culture medium alone. Subsequently, 10 μL of CCK-8 solution was added to each well, and the plates were incubated for 1–2 h. The absorbance of each well at 450 nm was measured using a full-wavelength enzyme labeler.

### 2.7. U(VI) Uptake and Release

In vitro U(VI) uptake and release assays were conducted to investigate the uranyl removal efficiency of PS at the cellular level. Exponentially growing NRK-52E cells were simultaneously exposed to 40 μg/mL UO_2_(CH_3_COO)_2_·2H_2_O and 5 μg/mL of the chelator. Groups exposed to U(VI) only were treated with UO_2_(CH_3_COO)_2_·2H_2_O. Additionally, control groups not exposed to U(VI) were cultured in a medium without treatment. After a 48 h incubation period, the cultured NRK-52E cells were washed with phosphate-buffered saline (PBS), trypsinized to detach them from the culture vessel, and counted using a cell counter. The cells were then subjected to microwave-assisted digestion and diluted with 5% nitric acid. The samples’ U(VI) content was subsequently determined using ICP-MS. The measurements were used to determine the concentration of ^238^U(VI) in each sample, where the intracellular ^238^U(VI) content was expressed in nanograms per 10^6^ cells [21].

However, because NRK-52E cells were cultured in a medium containing uranium throughout the experiments, the primary mode of action of the ligand, whether it blocks uranium uptake by the cells or promotes the release of uranium from the cells, remains uncertain. To accurately assess the chelator’s effect on promoting the release of uranium, we developed an additional in vitro method. This method involves the detection of U(VI) release from NRK-52E cells [21]. Initially, the cells were treated with a 40 μg/mL UO_2_(CH_3_COO)_2_·2H_2_O solution. The uranium-containing medium was replaced with a solution containing 5 μg/mL of the chelator. The post-processing techniques and subsequent computational methods used in our study are intended to be consistent with established models of immediate drug delivery and to ensure that our findings are consistent with current scientific understanding.

### 2.8. Statistical Analysis

In vitro, experimental data were obtained from no fewer than three independent experiments, with each experiment including triplicate measurements. All data are presented as the mean ± standard deviation (SD). Statistical analysis was conducted using nonlinear regression (curve fit) and one-way analysis of variance (ANOVA), as implemented in GraphPad Prism version 9.5.0. A coefficient of determination (R^2^) value greater than 0.9 indicates a high degree of correspondence between the experimental data and the fitted function. Statistical significance was defined as *p* < 0.05.

## 3. Results

### 3.1. Characterizations

**Figure 2:**
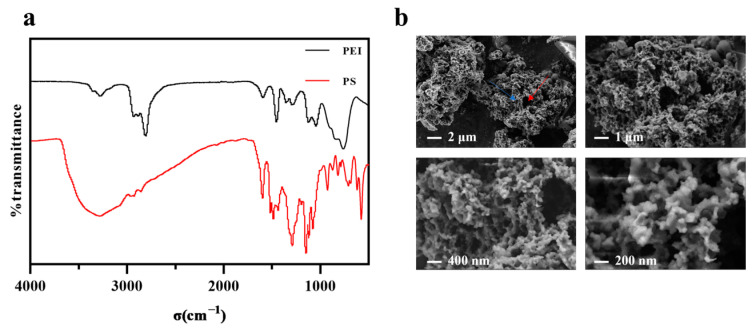
(**a**) FT-IR of PEI (black) and PS (red) highlighting characteristic vibrational bands; (**b**) SEM micrographs of PS at varying magnifications (red arrows indicate macroporous voids; blue arrows indicate surface defects).

The FT-IR (Figure 2a) analysis confirms the successful synthesis of the PS compound. For the PEI precursor (black trace), the broad band at 3278 cm^−1^ corresponds to the N–H stretching vibration of primary and secondary amines, the sharp absorption at 756 cm^−1^ to the out-of-plane N–H bending mode, and the doublet at 2936/2804 cm^−1^ to aliphatic C–H stretching of the –CH_2_– backbone. In PS (red trace), the broad absorption band at 3270 cm^−1^ is attributed to the O-H stretching vibration of the para-substituted phenolic hydroxyl groups, with peak broadening and frequency reduction resulting from intramolecular hydrogen bonding. The sharp peak at 1608 cm^−1^ corresponds to the C=C skeletal vibration of the aromatic ring, consistent with its para-substitution pattern. Distinctive absorptions at 1293 cm^−1^ and 1145 cm^−1^ are assigned to the asymmetric and symmetric stretching vibrations of the sulfonyl group (-SO_2_-), respectively. The S-N stretching vibration observed at 1070 cm^−1^ further corroborates the formation of the sulfonamide (-SO_2_-NH-) moiety. The doublet at 2940 cm^−1^ and 2856 cm^−1^ arises from C–H stretching vibrations of the aliphatic chain, while the multiplet spanning 1429–1528 cm^−1^ encompasses overlapping C=C aromatic vibrations and C-H in-plane bending modes. Notably, the significant attenuation of the N-H bending vibration (shifted from 756 cm^−1^ in PEI to 710 cm^−1^ in PS) provides direct evidence for the integration of the amino group into the sulfonamide linkage. Collectively, the emergence of sulfonamide-specific infrared signatures (1293, 1145, and 1070 cm^−1^) and the suppression of N-H bending confirm the structural integrity of PS, aligning with the designed molecular architecture. Figure 2b displays SEM micrographs of PS across sequential magnifications (2 μm to 200 nm), revealing a hierarchical architecture with macroporous aggregates (>50 nm voids), submicron particles (200–400 nm), and nanoscale surface defects. ^1^H NMR spectra of PEI and PS (Figures S1 and S2) were acquired to verify the marked attenuation of free primary/secondary amine signals (δ 2.4–2.8 ppm) and the appearance of aromatic protons (δ 6.84–7.30 ppm), confirming successful conjugation and demethylation.

### 3.2. Adsorption of Uranium

**Figure 3:**
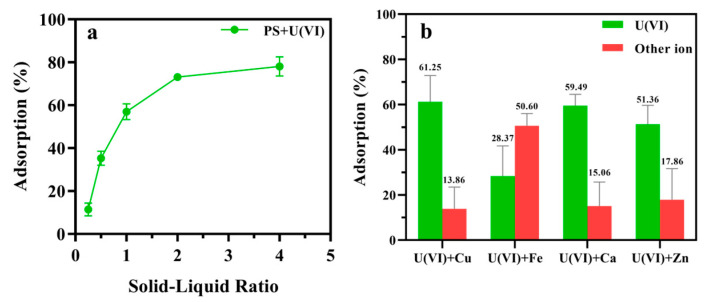
(**a**) solid–liquid ratios on adsorption of U(VI); (**b**) selectivity of PS toward UO_2_^2+^ ion (50 mg/L) and individual interfering ion (250 mg/L) in water solution. Bars indicate SD, n = 3 samples.

The *experimental* results demonstrate concentration-dependent uranium adsorption characteristics and ion-specific selectivity of PS. As shown in Figure 3a, uranium removal efficiency exhibited a progressive enhancement with increasing solid–liquid ratio, rising from 11.40% at 0.5 mg/mL to 78.08% at 4 mg/mL, with distinct saturation behavior observed beyond this threshold. Competitive adsorption studies (Figure 3b) revealed PS’s preferential uranium affinity across multi-ion systems, achieving 61.25% UO_2_^2+^ removal in Cu^2+^-containing solutions despite concurrent 13.86% copper adsorption. While Fe^3+^ competition resulted in reduced uranium removal (28.37%), this primarily reflects PS’s intrinsic iron-chelating functionality rather than compromised uranium selectivity, as evidenced by 50.60% Fe^3+^ adsorption attributable to structural iron-binding motifs. Notably, uranium removal consistently dominated over competitor ions in other systems, particularly in Ca^2+^-containing solutions (59.49% U vs. 15.06% Ca^2+^ removal) and Zn^2+^ environments (51.36% U vs. 17.86% Zn^2+^ removal), confirming the material’s engineered specificity for uranyl species.

### 3.3. Cytotoxicity Assay

**Figure 4:**
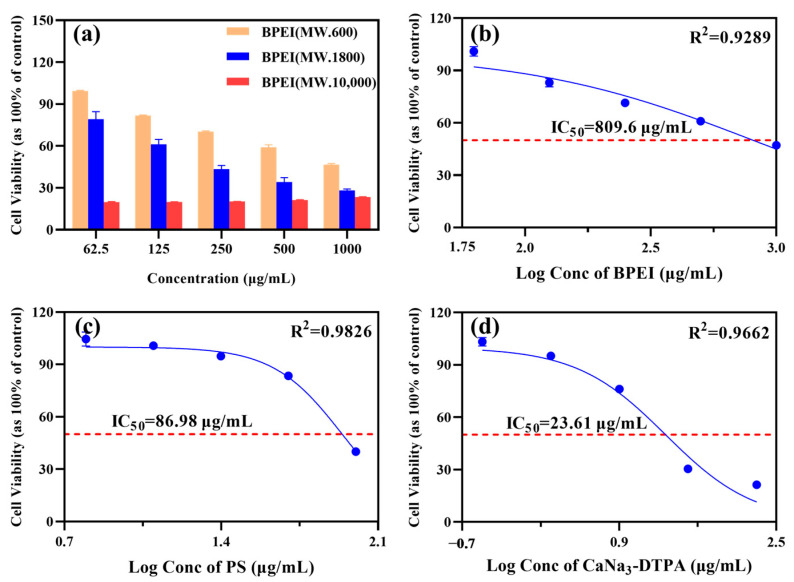
Dose-dependent effects on NRK-52E cell viability following treatment with (**a**) PEIs of varying molecular weights, (**b**) PEI, (**c**) PS, and (**d**) CaNa_3_-DTPA. Bars indicate SD, n = 3 samples. Blue solid lines, fitted dose–response curves; red dashed lines, IC_50_ values.

The molecular weight-dependent biocompatibility of PEI was established through cytotoxicity screening (Figure 4a), where systematic evaluation of three variants (600, 1800, and 10,000 Da) across 62.5–1000 μg/mL revealed an inverse correlation between molecular weight and renal cell viability. The 600 Da PEI demonstrated superior biosafety with an IC_50_ of 809.6 μg/mL (R^2^ = 0.9289) (Figure 4b), maintaining >80% viability at 1000 μg/mL, while intermediate (1800 Da) and high (10,000 Da) molecular weight counterparts exhibited 18.41% and 23.21% greater cytotoxicity, respectively. This molecular weight-dependent toxicity profile scientifically validates the selection of low-MW PEI (600 Da) as the optimal precursor for PS synthesis, ensuring maximal safety margins for subsequent therapeutic applications. Building on this molecular weight optimization, renal biosafety assessment of uranium decorporation agents showed that the 600 Da PEI precursor exhibited exceptional biocompatibility, whereas its structurally modified derivative PS displayed moderate cytotoxicity (IC_50_ = 86.98 μg/mL, R^2^ = 0.9826) (Figure 4c) within 6.25–100 μg/mL, representing a 9.3-fold tolerance reduction. In contrast, the positive control CaNa_3_-DTPA demonstrated substantially higher cytotoxicity (IC_50_ = 23.61 μg/mL, R^2^ = 0.9662) (Figure 4d), with PS maintaining 3.7-fold greater viability at equivalent concentrations.

The logarithmic concentration-response curves revealed strong correlations for all compounds (R^2^ > 0.90), with PS maintaining >60% cell viability across its entire test range versus <40% survival for CaNa_3_-DTPA at corresponding concentrations. This comparative analysis confirms that while PS synthesis moderately increases cytotoxicity relative to native PEI, the derivative retains superior renal biosafety compared to current clinical standards, validating its potential as a uranium-chelating agent with reduced nephrotoxic risk.

### 3.4. Effect of Chelating Agents on U(VI)-Induced NRK-52E Cells Injury

**Figure 5:**
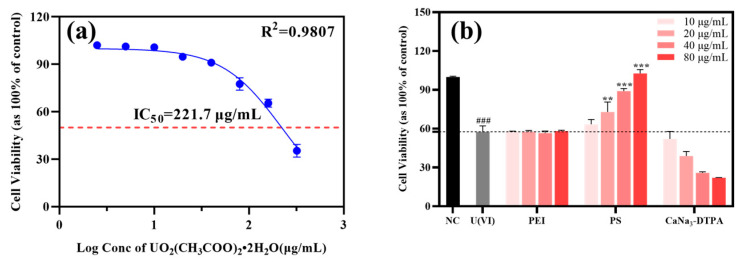
(**a**) Dose-dependent effects on NRK-52E cell growth rate following treatment with UO_2_(CH_3_COO)_2_·2H_2_O. (**b**) Effects of chelating agents on U(VI)-induced injury in NRK-52E cells. Blue solid lines, fitted dose–response curves; red dashed lines, IC_50_ values; black dashed lines, cell viability of the U(VI)-treated control. ### *p* < 0.001 compared to the control group, *** *p* < 0.001, ** *p* < 0.01 compared to the U(VI)-treated control. Bars indicate SD, n = 3 samples.

As shown in Figure 5a, NRK-52E cells were exposed to uranyl acetate dihydrate [UO_2_(CH_3_COO)_2_·2H_2_O] at concentrations ranging from 2.5 to 320 μg/mL for 48 h. Dose–response analysis revealed an IC_50_ value of 221.7 μg/mL (R^2^ = 0.9807), prompting the selection of 200 μg/mL for subsequent model construction. Figure 5b demonstrates that U(VI)-exposed controls exhibited 57.56 ± 4.68% cell viability, whereas co-treatment with the chelator PS at 20, 40, and 80 μg/mL significantly increased viability to 72.93 ± 7.58%, 89.01 ± 1.99%, and 102.73 ± 2.93%, respectively (*p* < 0.05 for all concentrations vs. U(VI) group). Notably, PS outperformed CaNa_3_-DTPA (at equivalent doses), while the precursor PEI exhibited no protective effect across the administered dose range of 10–80 μg/mL, with cell viability remaining stable between 57.71 ± 0.38% and 58.21 ± 0.60%. These results indicate that PS-mediated uranium chelation reduces free U(VI) bioavailability, thereby attenuating cytotoxicity and restoring cellular homeostasis more effectively than clinically used DTPA.

### 3.5. U(VI) Uptake and Release

Toxicity and decorporation effect are key evaluation criteria for actinide decorporation agents. This study evaluated the combined cytotoxicity of a newly synthesized chelator with uranyl ions and compared them with the commercially available decorporation agent CaNa_3_-DTPA. Similarly, we used NRK-52E for cellular evaluation. Based on the cytotoxicity data from Figure 5a, which evaluated the effects of uranyl ions on cell viability, we determined that a concentration of 40 μg/mL UO_2_(CH_3_COO)_2_·2H_2_O would be appropriate for subsequent experiments investigating combined toxicity and U(VI) uptake and release effects.

**Figure 6:**
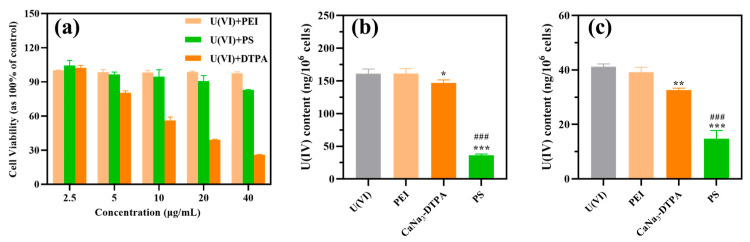
(**a**) Dosage-dependent growth rate of NRK-52E cells treated with U[(VI), 40 μg/mL] + PEI, U[(VI), 40 μg/mL] + PS, and U[(VI), 40 μg/mL] + CaNa_3_-DTPA; (**b**) Immediate administration; and (**c**) Delayed administration effects of PEI (5 μg/mL), PS (5 μg/mL), and CaNa_3_-DTPA (5 μg/mL) on the uptake and release of U(VI) in NRK-52E cells exposed to U(VI) (40 μg/mL). *** *p* < 0.001, ** *p* < 0.01, * *p* < 0.05 compared to the U(VI)-treated control. ### *p* < 0.001 compared to the CaNa_3_-DTPA. Bars indicate SD, n = 3 samples.

Therefore, a comprehensive toxicity assay of U(VI) and chelating agents was performed by adding 40 μg/mL UO_2_(CH_3_COO)_2_·2H_2_O and different concentrations of chelating agents ranging from 2.5 to 40.0 μg/mL. As depicted in Figure 6a, compound PS displayed superior-combined cytotoxicity profiles at concentrations ranging from 2.5 to 40 μg/mL, with lower cytotoxicity levels than those observed for CaNa_3_-DTPA. Notably, the cytotoxicity in the CaNa_3_-DTPA treatment group was pronounced at higher concentrations, resulting in a cell survival rate below 60% at doses between 10~40 μg/mL. This marked difference in overall cytotoxicity, compared to the chelating agent PS at equivalent concentrations, led us to exclude this concentration range for cellular decorporation studies.

However, at a concentration of 5 μg/mL, the cell survival rate in the CaNa_3_-DTPA group was 80.53 ± 1.67%, which was comparable to the cytotoxicity of chelator PS at the same concentration, all exhibiting low cytotoxicity. This similarity in cytotoxicity at concentrations below 5 μg/mL suggests that chelator PS has a similar safety profile to CaNa_3_-DTPA. Consequently, a concentration of 5 μg/mL was selected as the cellular decorporation evaluation concentration for assessing the decorporation efficacy of the chelating agents. To determine the principal mode of action of the chelating agent in the context of uranium toxicity, we established two distinct delivery models (immediate and delayed). The first model involved immediate administration of the chelating agent to assess its ability to block uranium uptake by cells. The second model was designed to examine the agent’s efficacy in promoting the release of uranium from cells when delivered after an initial exposure period. These models provided a framework for understanding the agent’s role in mitigating uranium entry or enhancing its elimination from cells.

As demonstrated in Figure 6b,c, compound PS exhibited significant uranium removal efficacy in both administration models. Under immediate administration conditions, PS reduced U(VI) levels by 77.37% compared to the U(VI)-treated group, while CaNa_3_-DTPA achieved only an 8.7% reduction under identical parameters. In delayed administration scenarios, PS maintained superior performance with a 64.18% decrease in U(VI) content relative to the control group, whereas CaNa_3_-DTPA showed limited efficacy with a 20.86% reduction. These results conclusively demonstrate that PS outperforms the positive control drug CaNa_3_-DTPA in uranium elimination efficiency under equivalent mass concentration dosing conditions, regardless of administration timing (immediate or delayed).

## 4. Discussion

This study successfully developed and evaluated a novel polymeric chelator, PS, based on low-molecular-weight BPEI modified with 3,4-dimethoxybenzenesulfonyl groups, for enhanced uranium decorporation. The design rationale centered on leveraging the high chelation capacity of BPEI’s three-dimensional architecture and integrating sulfonamide functionalities to improve uranyl coordination and cellular permeability. Key findings demonstrate that PS exhibits superior uranium adsorption efficiency, selectivity, and biocompatibility compared to the positive control drug CaNa_3_-DTPA, addressing critical limitations of current decorporation therapies.

Compared with traditional small molecule chelators like CaNa_3_-DTPA and EDTA, PS shows significant advantages in uranium adsorption capacity and selectivity. For example, in a study by Leydier et al. [4], EDTA- and DTPA-modified ligands were investigated as sequestering agents for uranyl decorporation. Their results showed that these traditional chelators had reduced uranium adsorption capacity in seawater media due to the interference of competing ions. In contrast, PS demonstrated a high uranium adsorption capacity of 78.08% at a concentration of 4 mg/mL, with significant selectivity over competing ions such as Ca^2+^, Zn^2+^, and Cu^2+^. This superior adsorption capacity and selectivity of PS can be attributed to its unique design combining the high chelation capacity of BPEI and the high selectivity of siderophores.

In terms of biocompatibility, PS also outperforms traditional chelators. As mentioned in the study by Muller et al. [6], the use of DTPA for uranium decorporation is prone to cause kidney damage and other toxic side effects. However, cytotoxicity assays in our study revealed that PS had an IC_50_ of 86.98 μg/mL, which is 3.7-fold higher than that of CaNa_3_-DTPA (23.61 μg/mL). This enhanced biosafety likely stems from the branched BPEI backbone, which mitigates membrane disruption by reducing surface charge density. For instance, Lahrouch et al. [8] reported that methylcarboxylated polyethyleneimine (PEI-MC) exhibited good biocompatibility and was effective as a uranium decorporation agent. Our PS, with its optimized molecular weight and introduced sulfonamide moieties, further improves the balance between chelation efficacy and biocompatibility.

Moreover, PS shows better performance in reducing intracellular uranium levels compared to traditional chelators. In a uranium exposure model (200 μg/mL), PS significantly improved cell survival rates and reduced intracellular uranium levels by 77.37% (immediate administration) and 64.18% (delayed administration). In comparison, CaNa_3_-DTPA achieved only an 8.7% reduction in immediate administration and a 20.86% reduction in delayed administration scenarios. This indicates that PS is more effective in mitigating the hazards of uranium exposure at the cellular level. The observed selectivity of PS for uranium over competing ions, particularly in Ca^2+^-rich environments, is critical for clinical translation, as calcium displacement by traditional chelators like DTPA often exacerbates nephrotoxicity [22]. Furthermore, the hierarchical porosity of PS, as revealed by SEM, likely facilitates intracellular uranium sequestration through enhanced diffusion and surface interactions [23]. The reduced cytotoxicity of low-MW BPEI (600 Da) compared to higher-MW variants aligns with previous reports attributing toxicity to excessive electrostatic interactions with cellular membranes [24]. By optimizing molecular weight and introducing sulfonamide moieties, this study achieves a balance between chelation efficacy and biocompatibility, a critical advancement in polymeric chelator design.

Despite the promising results, this study has certain limitations. The evaluations were conducted exclusively in vitro, and future research should validate the efficacy and safety of PS in animal models to assess pharmacokinetics, biodistribution, and long-term safety. This will be crucial for the clinical translation of PS as a potential uranium decorporation agent. Additionally, the competitive adsorption experiments focused on individual ions rather than complex biological matrices, which may better simulate in vivo conditions. Future studies should explore PS’s decorporation efficacy in vivo, investigate its renal clearance profile, and evaluate potential off-target interactions with essential metal ions.

## 5. Conclusions

We have translated the concept of a “polymer–siderophore hybrid” into a readily accessible molecule, PS, that achieves gram-scale production in two straightforward steps without costly catalysts or chromatography. The material displays record-high uranium removal under mild, near-physiological conditions—78% extraction at 4 mg/mL within 24 h—and retains more than half of this performance even when challenged by a five-fold excess of competing earth-abundant cations, a scenario typical of seawater or blood serum. Equally important, its cytotoxicity threshold lies 3.7-fold above that of the clinical standard CaNa_3_-DTPA, while a single 5 µg/mL dose lowers intracellular uranium by 64–77% irrespective of whether administration is immediate or delayed. These metrics collectively indicate that PS can operate effectively at concentrations at least one order of magnitude below those required by current chelators, sharply reducing the risk of nephrotoxicity and easing dosing regimens. Because the underlying branched PEI scaffold is already produced industrially and the catechol-sulfonamide ligand is derived from inexpensive commodity chemicals, the route is inherently scalable; the absence of precious metals or protecting-group manipulations keeps projected costs well below those of other advanced adsorbents or decorporation agents. Consequently, PS is positioned to bridge laboratory discovery with real-world deployment—whether as packed-bed cartridges for groundwater treatment, membrane coatings for seawater mining, or injectable therapeutics for accidental exposure—offering a single platform that addresses both environmental cleanup and human health protection without the compromises that have so far limited existing technologies.

## Data Availability

The original contributions presented in this study are included in the article/Supplementary Material. Further inquiries can be directed to the corresponding author.

## Supplementary Materials

The following supporting information can be downloaded at the following address: https://www.mdpi.com/article/10.3390/polym17162256/s1. Figure S1. ^1^H-NMR spectrum of PEI (600 Da) in DMSO-d_6_. Figure S2. ^1^H-NMR spectrum of PS in DMSO-d_6_.
