# Supplementary material for: 3,4-Dihydroxybenzenesulfonyl-Functionalized Polyethyleneimine for Uranium Chelation
Source: Polymers (Basel). 2025 Aug 21;17(16):2256. doi: 10.3390/polym17162256 (PMC12389725; doi:10.3390/polym17162256)
Supplement: Supplementary file 1 [file polymers-17-02256-s001.zip › polymers-3749362-supplementary.pdf]

Supplementary Material

Section 1: ^{1}H-NMR Spectra of PEI (600 Da) and PS.

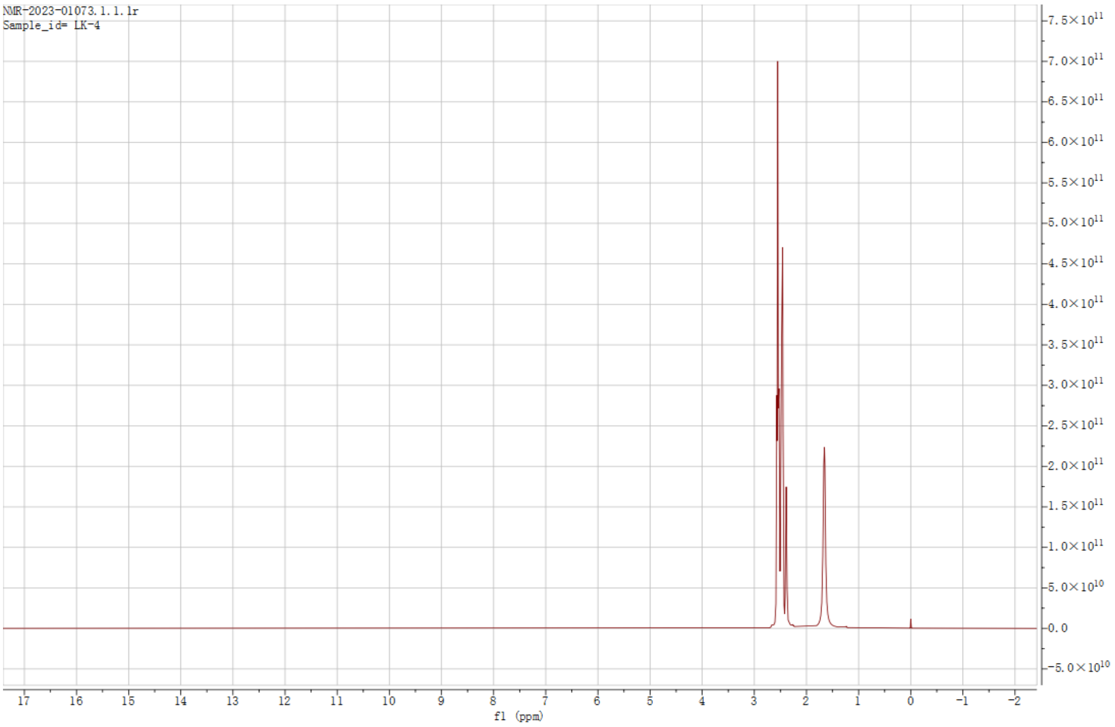

Figure S1. ^{1}H-NMR spectrum of PEI (600 Da) in DMSO-d_6.

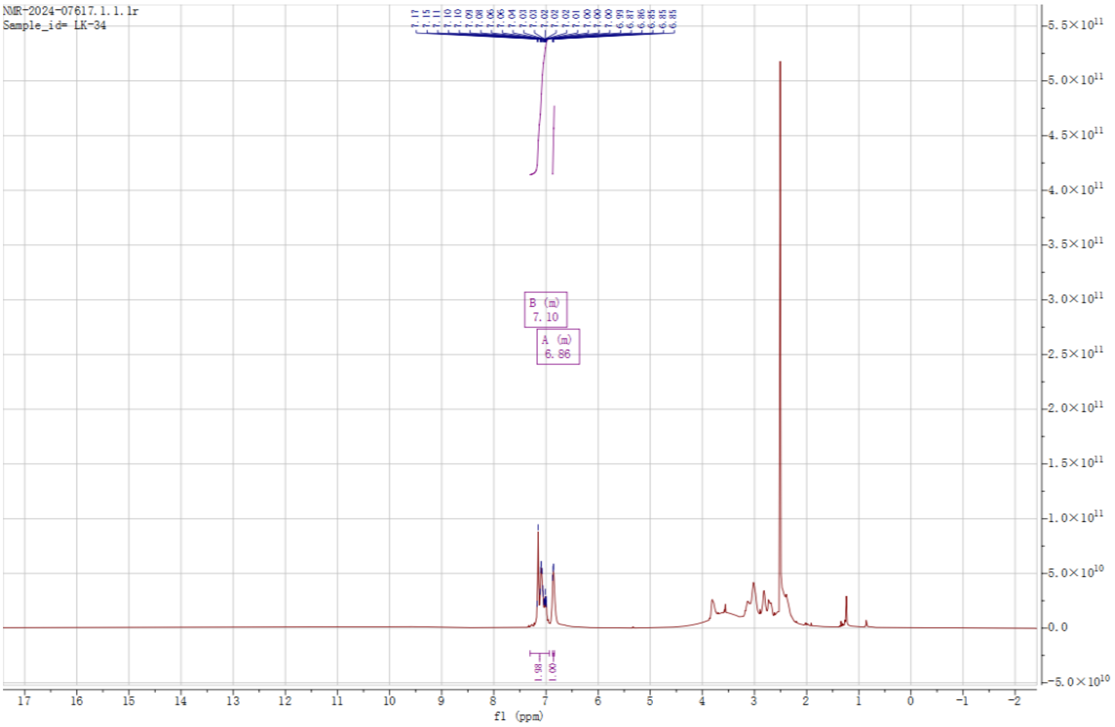

Figure S2. ^{1}H-NMR spectrum of PS in DMSO-d_6.
